# β‑Caryophyllene and Graphene Oxide: A Novel Approach for Managing *Fusarium* Wilt in *Cyclamen* spp.

**DOI:** 10.1021/acsomega.5c06285

**Published:** 2025-10-13

**Authors:** Andre May, Marcia R. Assalin, Bernardo A. Halfeld-Vieira, Kátia L. Nechet, Eunice R. Batista, Helio D. Quevedo, Marley M. Tavares, Claudio M. Jonsson, Sonia C. N. Queiroz

**Affiliations:** Embrapa Environment, Brazilian Agricultural Research Corporation, Rodv. SP 340, Km 127,5, Jaguariúna, SP CEP 13918-110, Brazil

## Abstract

Cyclamen (*Cyclamen* spp.) is a widely cultivated ornamental plant. Among the soil-borne pathogens affecting cyclamen, *Fusarium* wilt, caused by *Fusarium oxysporum* f. sp. *cyclaminis*, is one of the most significant phytosanitary challenges. This study aimed to evaluate the antifungal potential of graphene oxide and β-caryophyllene, separately and in combination, applied either by spraying or through endotherapy (bulb injection), to control *Fusarium* wilt in cyclamen. The combined application of these antifungal agents proved more effective, resulting in 40–60% mortality of infected plants, compared to 100% mortality in the untreated control group. A sorption study of fusaric acid on graphene oxide was also conducted to better understand its antifungal activity, along with an ecotoxicological assessment of β-caryophyllene to evaluate its environmental safety. Overall, the strong synergistic effect between graphene oxide and β-caryophyllene against *Fusarium oxysporum* f. sp. *cyclaminis* highlights their potential use in plant protection and supports the advancement of sustainable agricultural practices.

## Graphical abstract

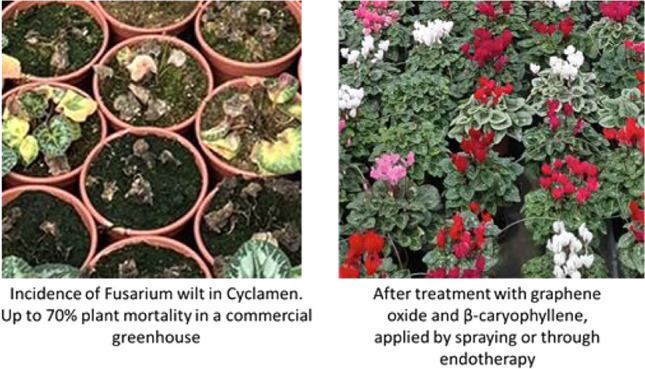

## 1 Introduction

Cyclamen (*Cyclamen* spp.), a member of the Myrsinaceae family (formerly Primulaceae), is widely valued as an ornamental plant due to its delicate flowers and attractive foliage. Native to Europe and the Mediterranean, its aesthetic appeal and commercial importance have led to its cultivation worldwide,^1–3^ with a strong presence in Holambra, Brazil. This region is renowned for its advanced production systems and is part of the largest ornamental flower market in the Americas.^4^ Despite its adaptability to diverse environmental conditions, cyclamen is highly susceptible to several pathogens, particularly soil-borne ones, which pose significant management challenges.^5,6^ Among these, *Fusarium* wilt, caused by *Fusarium oxysporum* f. sp. *cyclaminis* (Focy), is one of the most severe phytosanitary problems, with reports of up to 70% plant mortality in a commercial greenhouse of 4,000 plants in Holambra, Brazil.^7^ These pathogens employ infection strategies that include the production of toxins, causing foliar damage, apoptosis, and stomatal closure, ultimately impairing photosynthesis and ion transport.^8–11^ Given the economic importance of cyclamen in the ornamental plant industry, the development of effective and sustainable control strategies to mitigate the damage caused by these pathogens is crucial. Proper phytosanitary management is essential to preserve plant health, since pathogenic factors can severely hinder development.^5^ Consequently, ongoing studies on cyclamen’s resistance to specific pathogens are crucial to refine cultivation and conservation strategies, ensuring the durability and resilience of these plants in diverse environments. Biocontrol technologies have been proposed as alternatives to reduce agricultural crop residues, boosting research on natural compounds.^12–14^

β-Caryophyllene, a terpene found in plants of the Asteraceae and Cannabaceae families, has been identified as a key molecular signal in activating plant defenses against pathogens.^14–16^ *In vitro* studies have shown that β-caryophyllene can reduce the growth of *F. oxysporum* by up to 40%, without negatively impacting beneficial bacteria involved in growth promotion and nutrient cycling.^16^

In addition to natural plant defense mechanisms, graphene oxide has been studied as a novel antimicrobial agent against phytopathogenic bacteria and fungi. It has been shown to strongly inhibit the mycelial growth and spore germination of several fungal pathogens, including *Fusarium graminearum*, *Fusarium poae*, and *Fusarium oxysporum*.^17,18^ Due to its unique properties, graphene oxide can also protect against degradation and enhance the stability of beneficial substances, such as nutrients or fungicidal pesticides.^12,19,20^ Studies using reduced graphene oxide demonstrated its effectiveness in controlling *F. oxysporum* in plant roots, lowering wilt and root rot severity to less than 5% in tomato and pepper plants, without phytotoxic effects for approximately 70 days.^21^ The combined use of graphene oxide and β-caryophyllene, offering both fungicidal activity and benefits to the plant-microbiome system, represents an innovative approach.

Therefore, this study aimed to evaluate the potential of β-caryophyllene as a naturally derived fungicide and graphene oxide, separately and in combination, to assess their synergistic antifungal activity. Specifically, the reduction of *Fusarium* wilt incidence in cyclamen plants was investigated through *in vitro* and *in vivo* assays (via spraying and endotherapy [bulb injection]). Furthermore, to clarify the mechanism of graphene oxide against Focy, a sorption study of fusaric acid was conducted. Finally, given the potential of β-caryophyllene as a bioinput, an ecotoxicological assessment of this compound was performed.

## 2 Materials and Methods

### 2.1 Isolate of *Fusarium oxysporum* f. sp. *cyclaminis* (Focy) CMAA 1919

The isolate of *Fusarium oxysporum* f. sp. *cyclaminis* (Focy) CMAA 1919 was obtained from the Collection of Microorganisms of Agricultural and Environmental Importance at Embrapa Environment, Jaguariúna, São Paulo, Brazil. This isolate has had its whole genome sequenced (GenBank BioSample accession no. SAMN39596657, BioProject ID PRJNA1068603), and its pathogenicity was recently confirmed by ref. 7. Cultures were maintained on Potato Dextrose Agar (PDA) medium at 25 °C under a 12 h photoperiod for 15 days to produce colonies and spores for bioassays.

### 2.2 Plants

*Cyclamen* “Super Serie Verano Red Solar” or *Cyclamen* “Magenta” (*Cyclamen persicum* Mill.) seedlings were transplanted into 0.7 L pots filled with sifted soil supplemented with 10 g of NPK 10–10–10 fertilizer. Fifty days after transplantation, the plants were transferred to new containers. A second fertilization was applied after 60 days, using a slow-release fertilizer (3 g, Osmocote NPK 18–06–12). Irrigation was performed via a drip system three times daily, providing 50 to 100 mL of water per pot, depending on plant development. These plants were subsequently used in greenhouse experiments to evaluate the effects of treatments applied through different techniques.

### 2.3 Graphene Oxide and β-Caryophyllene

Graphene oxide was supplied by Padron & Padron (Araras, SP, Brazil). According to the manufacturer, the lateral size ranged from 1 to 12 μm, with a thickness of 0.55–1.2 nm (monolayer). The surface area is ∼500–1200 m^2^/g. The zeta potential (−36.3 mV) was measured using the microelectrophoresis technique with a ZetaSizer Nano Series instrument (Malvern Instruments).

### 2.4 *In Vitro* Biological Assays

#### 2.4.1 Evaluation of the Effect of Graphene Oxide on the Mycelial Growth of Focy

To evaluate the *in vitro* effect of graphene oxide on the mycelial growth of *Fusarium oxysporum* f. sp. *cyclaminis*, assays were conducted in Petri dishes. The experiment followed a completely randomized design with two treatments and five replications per treatment: Potato Dextrose Agar medium (PDA) and PDA supplemented with a 5% graphene oxide solution at a 1:20 ratio. A mycelial disc from an *F. oxysporum* colony (isolate CMAA 1919) was placed at the center of each Petri dish. The plates were incubated at 25 °C, and mycelial growth was assessed over a period of seven days.

#### 2.4.2 Evaluation of the Effect of β-Caryophyllene on the Mycelial Growth of Focy

The *in vitro* effect of volatile compounds from β-caryophyllene on the mycelial growth of *F. oxysporum* f. sp. *cyclaminis* was also evaluated in Petri dishes. The experiment followed a completely randomized design with two treatments and ten replications per treatment: PDA medium and PDA medium supplemented with 3 mL of a 1% β-caryophyllene solution in 2% Tween 80, applied to half of the dish. A mycelial disc from an *F. oxysporum* colony (isolate CMAA 1919) was placed in the center of the opposite partition. The dishes were incubated at 25 °C, and mycelial growth was monitored for 15 days.

### 2.5 *In Vivo* Biological Assays

In this study, pathogen inoculation with *F. oxysporum* was performed immediately after replanting, resulting in high mortality of cyclamen plants. The experiment was monitored until complete plant death in the control treatment, which occurred between 29 and 43 days, depending on plant genotype (“Magenta” or “Verano Red Solar”, respectively).

#### 2.5.1 Spraying on *Cyclamen* “Verano Red Solar” Plants

This experiment evaluated graphene oxide, β-caryophyllene, and their mixture as test solutions applied by spraying directly onto the bulbs of *Cyclamen* “Verano Red Solar” plants. For the greenhouse assay, the solutions consisted of: (i) 1% β-caryophyllene aqueous solution, (ii) 5% graphene oxide aqueous solution, and (iii) a 1% β-caryophyllene and 5% graphene oxide mixture in a 1:1 (v/v) ratio. The experiment comprised eight treatments, each with 15 replicates, arranged in a completely randomized block design, as shown in Table 1. Two absolute controls were included: one for plants inoculated with *F. oxysporum* f. sp. *cyclaminis* CMAA1919 (Focy) and one for noninoculated plants. The test solutions (3 mL per pot) were applied 50 days after transplantation. Inoculation with Focy was performed using a conidial suspension (5 mL at a concentration of 10^6^ conidia/mL) applied to the roots of previously injured plants immediately after replanting. Plant dry weight was determined at the onset of widespread yellowing, which marked the beginning of fusariosis in treated groups, just before plant death due to disease progression. The experiment continued until complete mortality of plants in the control treatment.

**Table 1:** Description of Treatments Evaluated in Each Experiment

Treatment	Description
T1	Control plants
T2	Focy-inoculated control plants
T3	Graphene oxide on plants
T4	β-Caryophyllene on plants
T5	Mixture of graphene oxide and β-caryophyllene (1:1, v/v) on plants
T6	Graphene oxide on Focy-inoculated plants
T7	β-caryophyllene on Focy-inoculated plants
T8	Mixture of graphene oxide and β-caryophyllene (1:1, v/v) on Focy-inoculated plants

#### 2.5.2 Endotherapeutic Application on *Cyclamen* “Magenta” Plants

This assay tested the endotherapeutic application of: (i) 1% β-caryophyllene aqueous solution with 2% Tween 80 (Scharlau, Spain), (ii) 5% graphene oxide aqueous solution with 2% Tween 80, and (iii) a 1% β-caryophyllene and 5% graphene oxide mixture in a 1:1 (v/v) ratio with 2% Tween 80. Test solutions (0.5 mL) were applied directly into the bulbs of *Cyclamen* “Magenta” plants (60 days post-transplant) using a syringe and needle. Treatments are listed in Table 1, with three replicates per treatment and two plants per replicate. Control treatments, inoculations with *F. oxysporum* f. sp. *cyclaminis*, and dry weight determinations were conducted as described in Section 2.5.1.

### 2.6 Adsorption Capacity of Fusaric Acid in Graphene Oxide

The adsorption capacity of graphene oxide was evaluated using fusaric acid solutions. Fusaric acid (CAS 536–69–6, C_10_H_13_NO_2_, 179.0946 g/mol) solutions were prepared at five concentrations ranging from 0.0050 to 3 μg/mL. Each sample consisted of 100 mg of graphene oxide combined with 5 mL of fusaric acid solution in a 20 mL glass vial sealed with a screw cap. Vials were agitated on a rotary shaker for 72 h to reach equilibrium. The equilibrium adsorption process was performed in triplicate for each concentration under isothermal conditions at 25 °C. After equilibration, 5 mL of suspension was filtered through a 0.22 μm disposable filter and analyzed using a liquid chromatography system (Waters Acquity Ultra Performance LC; Milford, MA, USA) coupled with a Triple-Quadrupole mass spectrometer (Quattro Premier XE; Milford, MA, USA) equipped with an electrospray ion source. Chromatographic separation was achieved using an Acquity UPLC BEH C18 column (100 mm × 2.1 mm; 1.7 μm, Waters) at 30 °C, with a 20 μL injection volume for samples and blanks. The mobile phase flow rate was 0.25 mL/min, consisting of 0.1% formic acid aqueous solution (solvent A) and acetonitrile/0.1% formic acid aqueous solution, 70:30, v/v (solvent B). The gradient program was: 0–3 min at 30% B, 3.1–5 min from 30% to 100% B, 5.1–8 min at 100% B, 8.1–9 min from 100% to 30% B, and 9.1–14 min at 30% B. Mass spectrometry was performed in positive ion mode under MRM conditions, monitoring transitions 179.9 > 133.9 and 179.9 > 161.93 for quantification and confirmation, respectively. Optimized instrument settings included: capillary voltage, 3.0 kV; cone voltage, 22 V; source temperature, 120 °C; desolvation temperature (nitrogen), 420 °C; desolvation gas flow (nitrogen), 500 L/h. The detection limit was 0.0005 μg/mL, with linearity confirmed between 0.0005 and 0.5 μg/mL (regression coefficient = 0.9969). Method reproducibility was satisfactory in all cases. Adsorption capacity (*q*_*e*_, ng/mg) was calculated according to Equation[Disp-formula eq1]:^22,23^

1 $$q_{e}=\left(C_{i}-C_{e}\right)/m\times V$$

where *V* (mL) is the solution volume, *m* (mg) is the mass of adsorbent, *C*_i_ (μg/mL) is the initial fusaric acid concentration, and *C*_e_ (μg/mL) is the equilibrium concentration.

### 2.7 GC/MS Analysis

After β-caryophyllene application, volatile compounds were monitored throughout the experimental period. For this study, the plant plots were sealed with plastic film to allow accumulation of volatile compounds. Sampling was performed using a manual SPME holder (Supelco-Aldrich, Bellefonte, PA, USA) equipped with a fused silica fiber coated with divinylbenzene/carboxen/polydimethylsiloxane (DVB/CAR/PDMS; 50/30 μm). The fiber was inserted into the sealed plant pot system for 15 min. Prior to sampling, all fibers were conditioned at 270 °C for 60 min, as recommended. The extracted compounds were analyzed using a gas chromatograph (Agilent 7890B) coupled with an Agilent 5977B single-quadrupole mass spectrometer. Separation was performed on an HP-5MSUI capillary column (30 m × 250 μm × 0.25 μm). The GC inlet was set at 250 °C in splitless mode, with high-purity helium (99.999%) as the carrier gas at a constant flow of 1.2 mL/min. The oven program was: 40 °C (1 min), ramped at 5 °C/min to 150 °C (1 min), followed by a 10 °C/min increase to 250 °C (held for 1 min). The solvent delay was 1 min, and the total run time was 35 min. The mass spectrometer operated in electron impact (EI) mode at 70 eV, scanning *m*/*z* 50–450. Data were processed with Agilent Mass Hunter Workstation and Unknowns Analysis software, version 10.0 (Agilent Technologies, Santa Clara, CA, USA). β-Caryophyllene was identified by mass spectra matching with the NIST Tandem Mass Spectral Library v.2.3 (NIST, Washington, DC, USA) and by comparison of retention times with an analytical standard.

### 2.8 Ecotoxicological Study of β-Caryophyllene

#### 2.8.1 Test Material

Test solutions containing β-caryophyllene (0.0, 0.01, 0.10, 1.00, 10.00, and 100.00 mg/L) were prepared from a commercial product (≥80%, Sigma-Aldrich), using Tween 20 Mol Bio grade (Crescent Chemical Co Inc., NY, USA) as a solubilization adjuvant.

#### 2.8.2 Assessment of Toxicity and Low-Risk Concentration

Dose–response studies were conducted according to the methodologies described by refs. 24, 25 , using *Raphidocelis subcapitata* (microalgae), *Lemna minor* (macrophyte), *Daphnia magna* (microcrustacean), *Artemia salina* (microcrustacean), and *Panagrolaimus* sp. (nematode). After exposure, effective concentrations causing 50% inhibition (EC50) were determined. EC50 values were calculated for 48 h (*Daphnia magna* and *Artemia salina*), 96 h (*Panagrolaimus* sp.), and 168 h (*Raphidocelis subcapitata* and *Lemna minor*). For the latter, phytotoxicity was assessed by measuring cell density and frond number, respectively. Confidence intervals (95%) were calculated using “Probit Analysis” in Statgraphics Centurion XVII, v. 1.17.04 (StatPoint Technologies). For phytotoxicity data, “Simple Regression” was applied using the same software. The hypothetical concentration of risk for 5% of species in a community (HC5) was estimated from a log–logistic distribution of the No Observed Effect Concentration (NOEC).^26–28^ HC5 was based on the lower confidence limit of the “Cumulative frequency vs Log NOEC” regression at a 50% certainty level^29,30^ using Statgraphics Centurion XVII software v. 1.17.04 (StatPoint Technologies). NOEC values were estimated as EC50/10 ^31^ or as the upper limit of the EC10 confidence interval (effective concentration by 10%).^32^

## 3 Results and Discussion

### 3.1 *In Vitro* Antifungal Activity Assays

No significant inhibition was observed in the graphene oxide-enriched medium. By the third day after subculturing, fungal growth was 6.9% lower in the graphene oxide treatment compared to the control. However, growth equalized over time, indicating graphene oxide was ineffective for fungal biocontrol under these assay conditions. According to Wang et al.,^33^ the antifungal effect of graphene oxide is concentration-dependent. At concentrations >250 mg/L, graphene oxide interferes with the synthesis of about 17 proteins in *Fusarium graminearum*, affecting mycelial growth, cell wall development, and stress response. It may also disrupt nutrient metabolism (e.g., glucose, succinate, citrate, GABA, glutamine, trehalose). In the present study, the graphene oxide concentration was about 56 times lower than that used by Wang et al.,^33^ which may explain the absence of fungicidal activity. In addition to the low concentration, discrepancies may also be related to differences between agar-based assays and the liquid media used by.^33^ It is noteworthy that increasing graphene oxide concentration could be economically unfeasible for field application due to associated material costs. El-Abeid et al.^21^ observed biocontrol of *F. oxysporum* on PDA medium using copper nanoparticles coated with reduced graphene oxide, where only 1 mg/L was sufficient for inhibition. This suggests that graphene oxide alone may be inadequate for fungicidal purposes, as demonstrated using graphene oxide in combination with a metal. Figure 1 illustrates the *in vitro* assay (duplicate plates) testing β-caryophyllene against *Fusarium oxysporum* f. sp. *cyclaminis* after seven days. Results were qualitative (visual inhibition assessment). Mycelial growth was completely inhibited by β-caryophyllene, confirming its strong antifungal potential through volatile action in a split-plate assay. In contrast, the control (PDA medium without β-caryophyllene) exhibited complete mycelial growth throughout the plate. The antifungal activity of terpenes has been reported previously. Hilgers et al.^16^ observed that direct β-caryophyllene exposure could inhibit *F. oxysporum* growth by up to 20%, with strain-dependent variation. These findings reinforce differences in the compound’s mechanism of action and support our findings.

**Figure 1:**
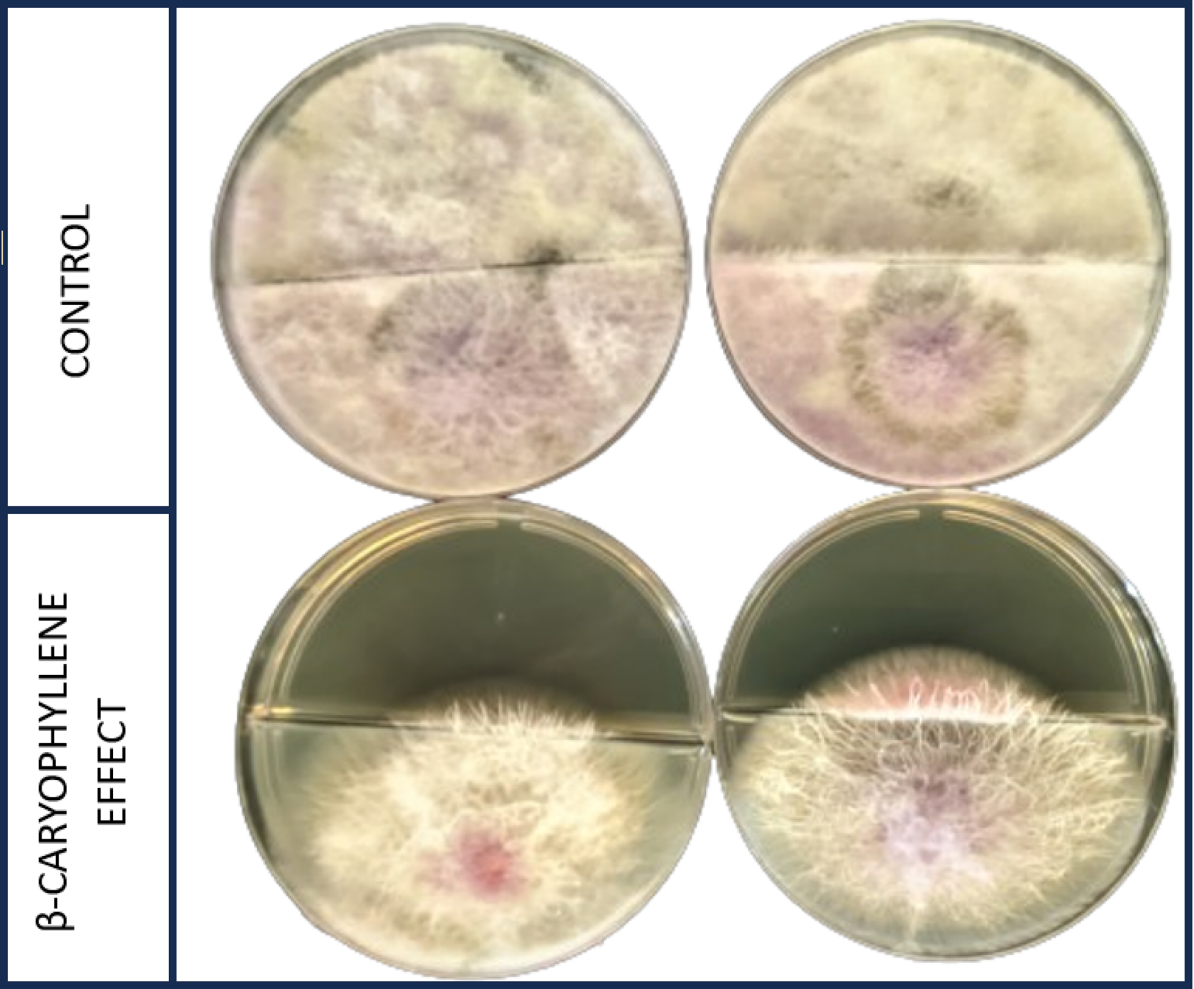
*In vitro* bioassay (duplicate plates) demonstrating the effects of β-caryophyllene volatile compounds on the mycelial growth of *Fusarium oxysporum* f. sp. *cyclaminis*, seven days post-treatment. The control (without β-caryophyllene) and the treatment (3% β-caryophyllene solution in the upper Petri dish partition) are shown.

### 3.2 Spray Application to *Cyclamen* “Verano Red Solar” Plants

Plants can emit and perceive a wide range of mono- and sesquiterpenes, which are well-known to function as signaling molecules in interspecific interactions.^34–36^ Frank et al.^37^ demonstrated that β-caryophyllene induces resistance in *Arabidopsis thaliana* against *Pseudomonas syringae* via jasmonic acid signaling. Field experiments have also shown that natural biological emissions of β-caryophyllene can induce resistance even in neighboring plants.

In our preliminary assays, we investigated the volatile organic compounds emitted by *Cyclamen* “Verano Red Solar” plants infested with *Fusarium oxysporum* f. sp. *cyclaminis* CMAA1919 (Focy). Figure 2 shows chromatograms of volatile organic compounds emitted by infested and noninfested plants. β-caryophyllene was identified at a retention time of 24.56 min (match factor: 96.91), but only in the chromatogram of Focy-infested plants. This compound is one of the biogenic volatile organic compounds emitted by infested cyclamen plants that is potentially associated with the induction of plant resistance. Therefore, we decided to supplement the plant with this compound to strengthen it and increase its resistance against this fungus. Because we had the analytical standard, we were able to confirm β-caryophyllene through retention time and NIST library correlation. After application, β-caryophyllene remained detectable for up to eight days when applied alone and for up to five days when combined with graphene oxide.

**Figure 2:**
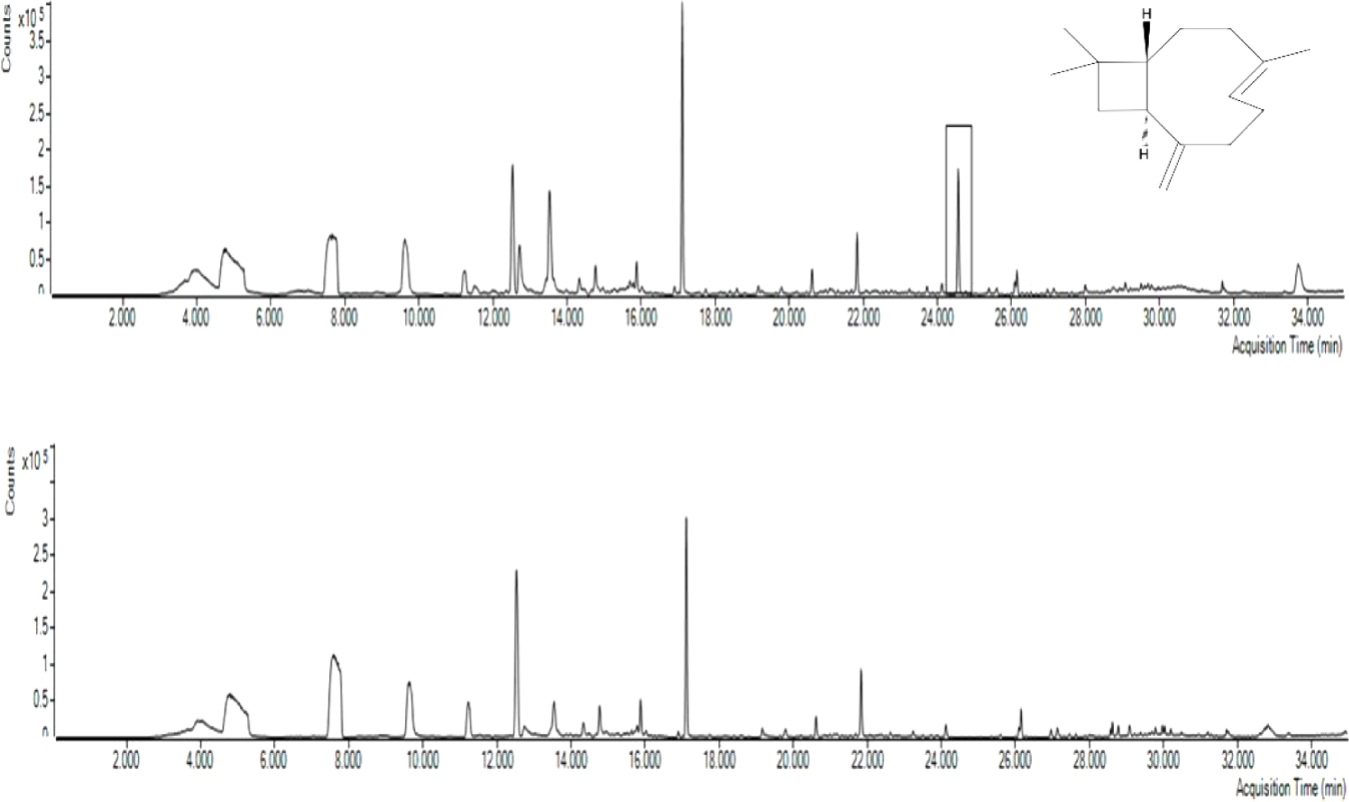
A. Chromatogram obtained for the analysis of volatile organic compounds emitted by plants inoculated with Focy. B. Chromatogram obtained for the analysis of volatile organic compounds emitted by plants without Focy.

The progression of *Fusarium* wilt incidence in cyclamen plants subjected to bulb/soil spray treatments is illustrated in Figures 3 and 4.

**Figure 3:**
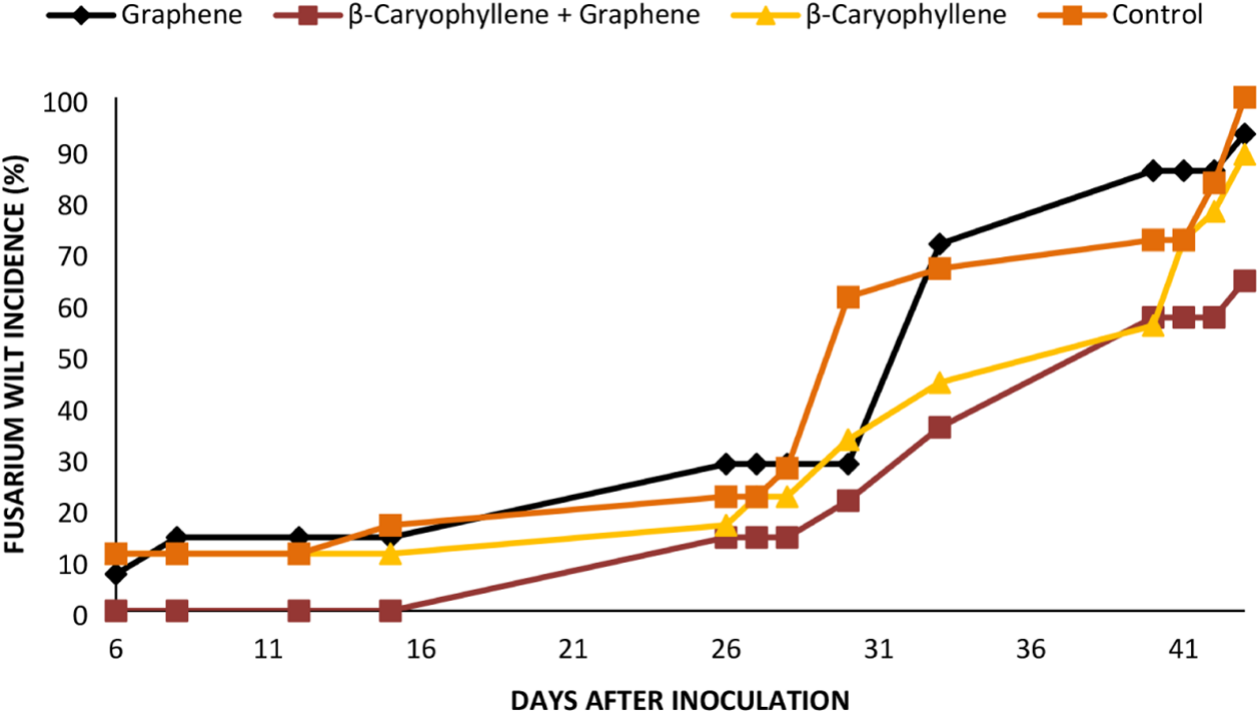
Progression of *Fusarium* wilt incidence in cyclamen plants after inoculation with *Fusarium oxysporum* f. sp. *cyclaminis* CMAA 1919. Control represents untreated plants inoculated with CMAA 1919.

**Figure 4:**
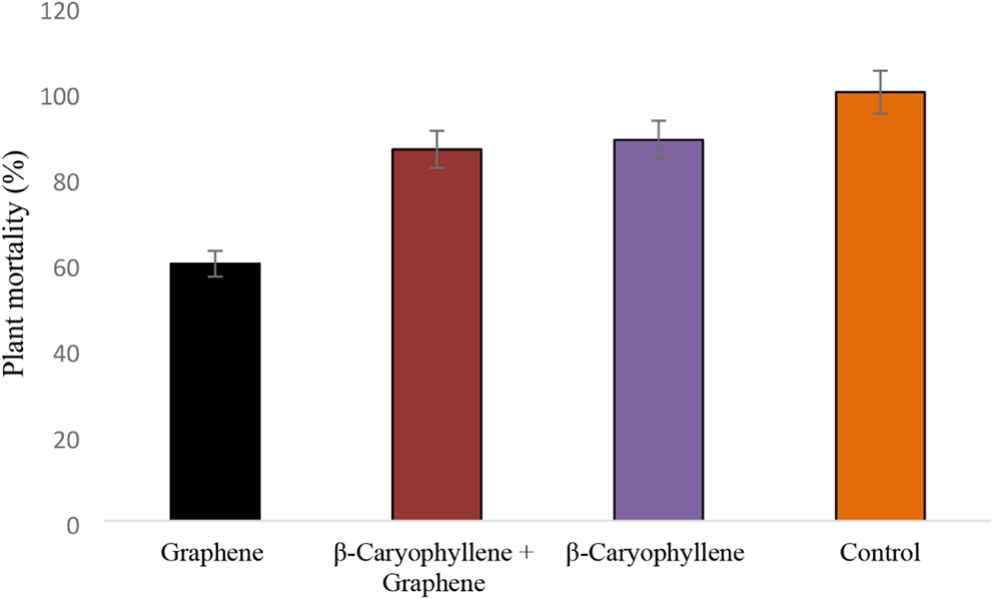
Mortality of cyclamen resulting from infection by *Fusarium oxysporum* f. sp. *cyclaminis*. Bars represent the standard error (5%).

The bioassay results confirmed the *in vitro* findings, highlighting β-caryophyllene as a potential inhibitory agent against *F. oxysporum* f. sp. *cyclaminis* and reinforcing its antifungal properties. Reduced plant mortality under β-caryophyllene treatments may be related to its ability to induce plant resistance (Figure 4). For all treatments except graphene oxide + β-caryophyllene, disease symptoms appeared six days postinoculation, with ∼10% mortality. In contrast, approximately 10% of plants treated with graphene oxide + β-caryophyllene only showed symptoms of *Fusarium* wilt only after 26 days. At 41 days, mortality was 100% for the control, 89% for graphene oxide, 87.5% for β-caryophyllene, and 60% for the combined treatment. It was observed that graphene oxide enhanced the toxicity of β-caryophyllene against the mycelial biomass due to the synergistic effect. We hypothesize that the increased fungicidal activity of β-caryophyllene when associated with graphene oxide is due to the ability of graphene oxide to adsorb β-caryophyllene. Graphene oxide can bind poorly soluble molecules through electrostatic attraction, hydrophobic interactions, and π–π stacking.^38,39^ The adsorption of β-caryophyllene provides protection against the factors that are responsible for its volatilization and degradation. In addition, the adsorption of β-caryophyllene on graphene oxide can improve the water dispersibility of the fungicidal compound, which may consequently improve its interactions with organisms.^17,40^ On the other hand, one of the infection strategies of *Fusarium oxysporum* includes the production of phytotoxins that cause foliar damage, apoptosis, and stomatal closure, all of which impair photosynthesis and ion transport. Fusaric acid, a secondary metabolite produced by *Fusarium* spp. species, is well-known for its strong phytotoxicity.^41^ Suggested mechanisms include the modification of cell membrane potential, inhibition of ATP synthesis, chelation of metal ions, and electrolyte leakage. However, the precise mechanism of fusaric acid phytotoxicity remains unknown.^42^ The rhizosphere microbiota constitutes the first layer of plant defense against soil-borne pathogens. Jin et al.^43^ demonstrated that fusaric acid produced by the pathogen *Fusarium oxysporum* f. sp. *lycopersici* can have distinct impacts on the modulation of the tomato rhizosphere microbiota. Fusaric acid can directly inhibit plant-beneficial bacteria, facilitating infection.^43^ We investigated the adsorption capacity of graphene oxide for fusaric acid (Figure 5). Our results show that 100 mg of graphene oxide almost completely adsorbed the tested doses of fusaric acid, reaching a maximum adsorption capacity (*q*) of 15.32 ng/mg, corresponding to 98% adsorption. Higher concentrations of fusaric acid would likely not be fully adsorbed as there would not be enough available active adsorption sites. Thus, by reducing the plant’s exposure to fusaric acid, graphene oxide may contribute to lowering disease incidence.

**Figure 5:**
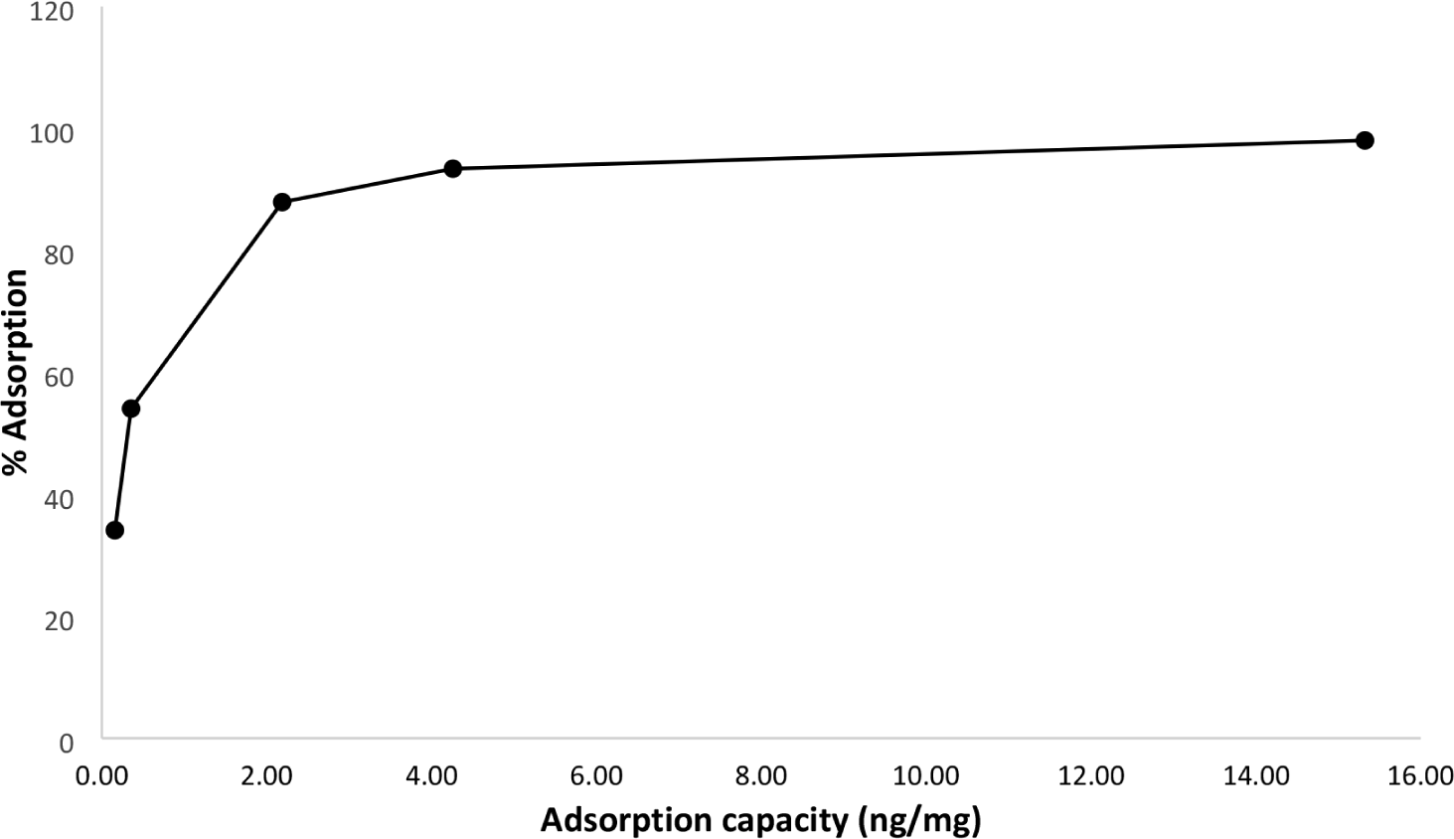
Effect of adsorption dose of fusaric acid by graphene oxide.

In addition to pathogen control and extended plant survival in pots, we observed another benefit of β-caryophyllene application in ornamental cultivation. Notably, application of β-caryophyllene, with or without graphene oxide, advanced flowering in 11% of plants compared to untreated controls. This effect is of commercial importance, as it combines disease protection with improved ornamental value. Table 2 presents the shoot dry weight data of aerial parts of *Cyclamen* “Verano Red Solar” plants under different treatments, showing increased biomass in plants treated with β-caryophyllene, graphene oxide (GO) and/or mixture of graphene oxide and β-caryophyllene (1:1, v/v) compared to controls.

**Table 2:** Shoot Dry Weight of *Cyclamen* “Verano Red Solar” Plants.^a^

Treatment	Description	Dry weight (g plant^–1^)
T1	Control plants	0.9 b
T2	Focy-inoculated control plants	0.9 b
T3	Graphene oxide on plants	1.5 a
T4	β-Caryophyllene on plants	1.7 a
T5	Mixture of graphene oxide and β-caryophyllene (1:1, v/v) on plants	1.6 a
T6	Graphene oxide on Focy-inoculated plants	0.7 b
T7	β-caryophyllene on Focy-inoculated plants	0.6 b
T8	Mixture of graphene oxide and β-caryophyllene (1:1, v/v) on Focy-inoculated plants	0.8 b
	**CV (%)**	40.83

a Means followed by the same letter do not differ from each other according to the Shapiro–Wilk test at 5% significance.

#### 3.2.1 Endotherapeutic Application on *Cyclamen* “Magenta”

Figure 6 illustrates the progression of *Fusarium* wilt incidence in cyclamen plants subjected to treatments applied through endotherapy. The incidence of the disease was first observed in the control and graphene oxide treatments. Eight days after inoculation, 70% mortality was recorded in the control plants and 30% in the plants treated with graphene oxide. After 15 days, plant mortality reached 100% in the control, 80% in graphene oxide, 60% in β-caryophyllene, and 40% in graphene oxide + β-caryophyllene (Figure 6). The treatment with graphene oxide stabilized the disease on the 13th day and maintained the same incidence until the end of the trial. Figure 7 highlights the synergistic effect of applying graphene oxide + β-caryophyllene (12 days after application) in plants inoculated with *Fusarium oxysporum* f. sp. *cyclaminis* CMAA 1919.

**Figure 6:**
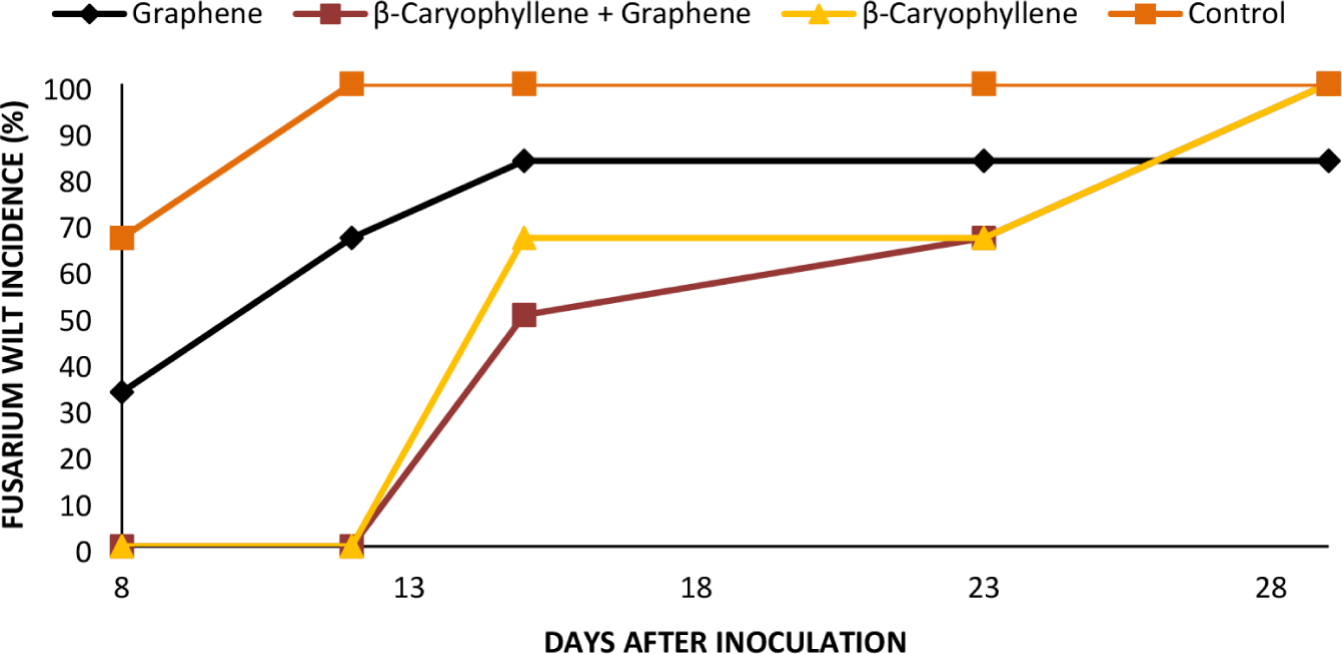
Incidence of *Fusarium* wilt on cyclamen plants after inoculation with *Fusarium oxysporum* f. sp. *cyclaminis* CMAA 1919. Control represents untreated plants inoculated with CMAA 1919.

**Figure 7:**
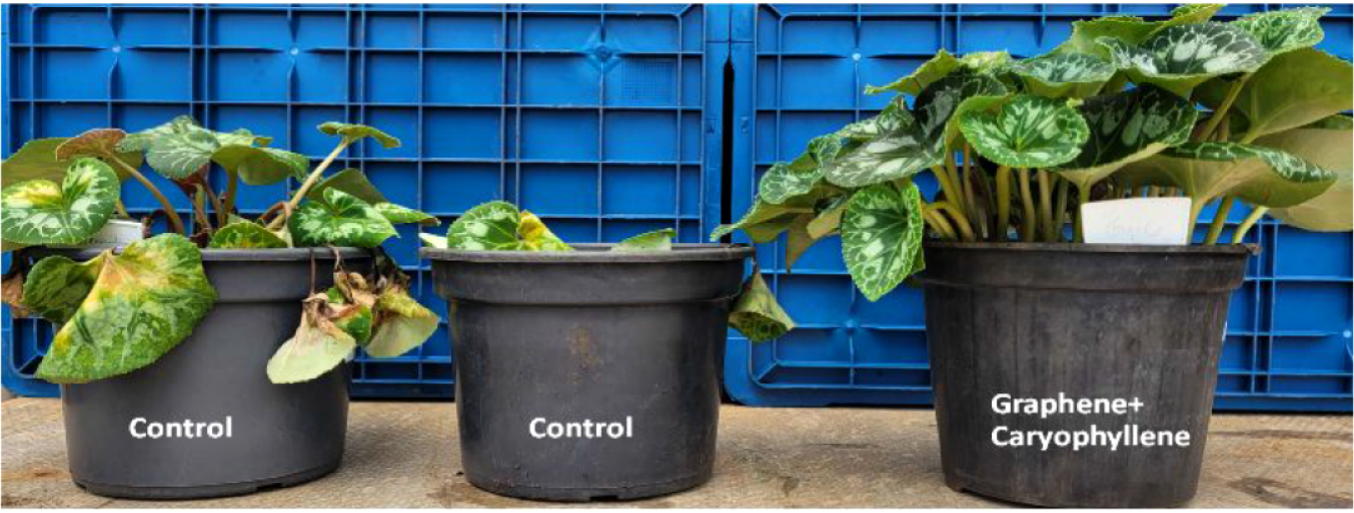
Illustrative photo showing the incidence of *Fusarium* wilt in *Cyclamen* “Magenta” plants 12 days after endotherapeutic application of the graphene oxide + β-caryophyllene mixture in plants inoculated with *Fusarium oxysporum* f. sp. *cyclaminis* CMAA 1919. The photo displays three pots: on the left—control (plants inoculated with *F. oxysporum* f. sp. *cyclaminis* without treatment), in the middle – treated with graphene oxide (GO), on the right – with the combined treatment (GO + β-caryophyllene).

Due to the rapid progression of the disease, it was not possible to observe the flowering effect in the “Magenta” genotype. However, at the end of the treatment, we examined whether there were differences in plant biomass.

Although there was a trend for treatments to show lower biomass compared to the control, no statistically significant difference was found to support these data (Figure 8). Therefore, we conclude that the application of β-caryophyllene or graphene oxide did not cause any harm to the productivity of plants infected by the pathogen. We also investigated the effect of applying β-caryophyllene and graphene oxide to plants without *Fusarium oxysporum* inoculation. We observed a significant increase in biomass in plants treated with β-caryophyllene and/or graphene oxide, indicating that the reduction in biomass (Figure 8) is linked to pathogen infection. This reveals that the application of both β-caryophyllene and graphene oxide has beneficial effects, improving growth factors that resulted in increased biomass in plants grown under these treatments.

**Figure 8:**
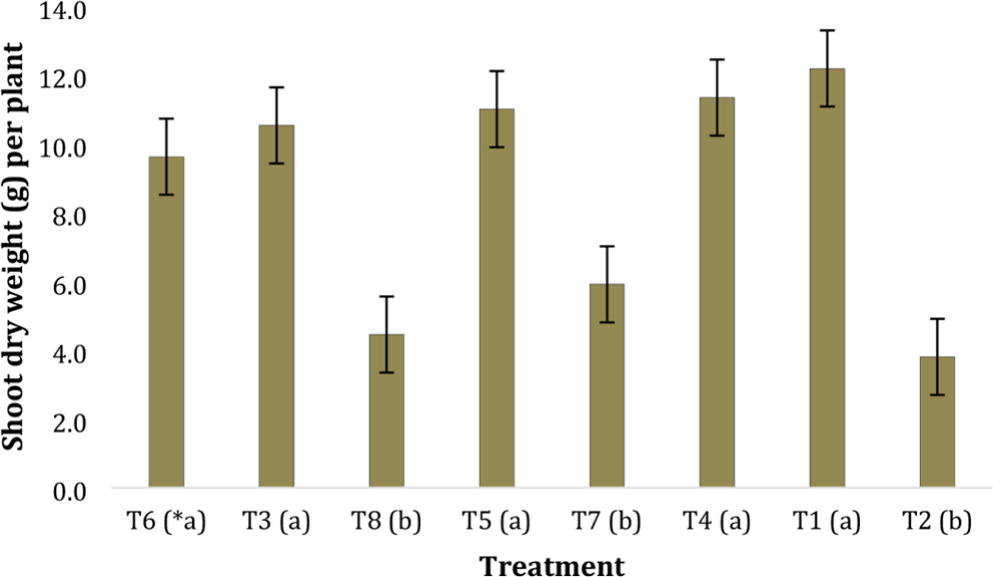
Shoot dry weight per plant (in grams) according to the treatments (treatments followed by the same letter (*) do not differ from each other according to the Scott–Knott test at a 5% probability level) (A: first assay, CV = 40.83%, B: second assay, CV = 14.41%). Treatments: T1 – Control plants; T2 – Control, *Fusarium oxysporum* f. sp. *cucumerinum* (Focy)-inoculated plants; T3 – Graphene oxide on plants; T4 – β-caryophyllene on plants; T5 – Mixture of graphene oxide and β-caryophyllene (1:1, v/v) on plants; T6 – Graphene oxide on Focy-inoculated plants; T7 – β-caryophyllene on Focy-inoculated plants; T8 – Mixture of graphene oxide and β-caryophyllene (1:1, v/v) on Focy-inoculated plants.

The proposed interaction between graphene oxide (GO) and β-caryophyllene is primarily adsorption-based, with GO acting as a carrier to stabilize the volatile β-caryophyllene. The presence of C=C double in β-caryophyllene bond can play a role through the π–π interactions. This adsorption protects β-caryophyllene, a volatile sesquiterpene, from volatilization and degradation by environmental factors (e.g., light, oxygen, heat), enhancing its antifungal efficacy in the combined treatment. Regarding the physicochemical properties of GO: its typical lateral size is 1–2 μm, thickness 0.55–1.2 nm; surface charge is negative (zeta potential −36 mV) due to deprotonated carboxyl groups; and surface area is high (∼500–1200 m^2^/g). These properties facilitate noncovalent binding with low-solubility molecules such as β-caryophyllene. In view of this, GO is likely to extend the half-life of β-caryophyllene by adsorbing it onto its surface.

### 3.3 Toxicity of β-Caryophyllene to Aquatic Organisms and Determination of a Low-Risk Concentration

This study evaluated the antifungal potential of graphene oxide and β-caryophyllene in controlling *Fusarium*. In this context, previous studies have raised concerns about the environmental risks associated with graphene oxide.^44,45^

Previous work conducted by our research group with a series of aquatic organisms, in analogy to the present study, indicated an HC5 value of 0.1 mg/L for graphene oxide, which corresponds to a concentration below which adverse effects are unlikely to occur during either short- or long-term exposure (Predicted No Effect Concentration – PNEC: 0.02–0.1 mg/L).^25^ In a study involving aquatic species from different trophic levels, Hong and Nowack^46^ reported results of a similar magnitude, with an average PNEC of ∼0.015 mg/L. The authors of both studies concluded that no risk is expected for aquatic ecosystems. Similar conclusions have also been reported by.^45^

According to Nemeth et al.,^47^ even under a worst-case scenario approach applied to two graphene oxide samples (derived from different graphite precursors), the tested materials did not pose an environmental risk. This study evaluated a range of organisms from different trophic levels (bacteria, protozoa, a freshwater microbial community, plants, and invertebrates) in aquatic environments.

In the present work, given the extremely low PNEC values reported for caryophyllene oxide (a β-caryophyllene metabolite)^48,49^ and the absence of risk values for β-caryophyllene in the literature, we aimed to determine limit concentrations of this compound to protect aquatic biota.

Because β-caryophyllene shows potential for use as a bioinput in agriculture, it is necessary to assess its risks to nontarget organisms through ecotoxicological testing. Therefore, this study was included here since, despite being a naturally occurring molecule, the environmental safety of its use must be evaluated. For this purpose, the following indicator organisms were tested: the microalga *Raphidocelis subcapitata*, the macrophyte *Lemna minor*, the microcrustacean *Daphnia magna*, the nematode *Panagrolaimus* sp., and the microcrustacean *Artemia salina*. The most sensitive organism to β-caryophyllene was *Raphidocelis subcapitata* (EC50–168 h = 0.97 (0.52–1.57) mg/L), with the compound classified as “highly toxic,” as the EC50–168 h value falls within the range 0.1–1.0 mg/L.^50,51^ *Artemia salina* and *Panagrolaimus* sp. showed EC50 values > 100 mg/L, classifying the compound as “practically non-toxic.” In *Lemna minor*, growth inhibition based on frond number, fresh weight, and total chlorophyll content led to a classification of “slightly toxic,” similar to *Daphnia magna* with respect to mobility. This classification corresponds to EC50 values in the range >10–100 mg/L.^51^

According to the product safety assessment sheet,^52^ β-caryophyllene is described as “not dangerous for the aquatic environment,” reporting EC50–48 h and EC50–72 h values of >0.17 mg/L and >0.033 mg/L for *Daphnia magna* and *Raphidocelis subcapitata*, respectively. These data, however, do not allow a clear conclusion regarding the compound’s risk. Nonetheless, Api et al.^48^ reported EC50 values of 0.329 and 0.281 mg/L in *Daphnia magna* and algae, respectively, for caryophyllene oxide. This compound, derived from β-caryophyllene metabolism, is used in cosmetic manufacturing.^49^ The authors estimated a PNEC for aquatic communities in the range of 0.00089–0.0281 μg/L.

The EC50 value for algal growth inhibition presented in this study was very close to that described by Api et al.^48^ for caryophyllene oxide. Those authors found similar levels of toxicity for both algae and microcrustaceans. Nevertheless, this was not observed in our results, where *Daphnia magna* proved much less sensitive (EC50–48 h = 49.07 mg/L). A possible explanation is a lower metabolization of β-caryophyllene to caryophyllene oxide in microcrustaceans compared with microalgae. Figure 9 demonstrates the log–logistic function of cumulative sensitivity based on NOEC values for different test organisms. The variability in sensitivity among species generated different NOEC values, allowing us to calculate an HC5 value of 0.026 mg/L. Applying safety factors of 1–5 to this value^53^ yields PNEC values in the range of 0.026–0.0052 mg/L.

**Figure 9:**
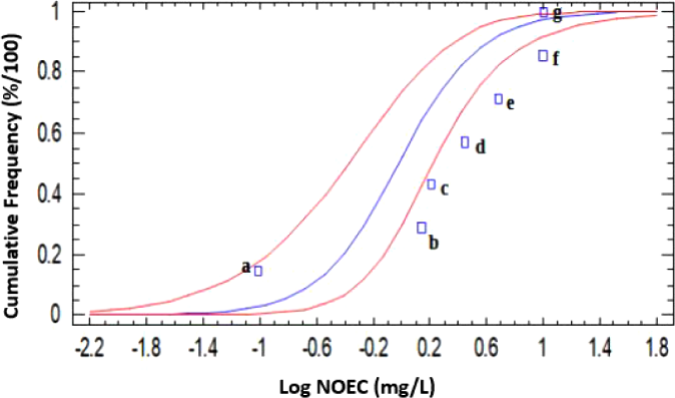
Sensitivity distribution curve based on No Observed Effect Concentration (NOEC) values of β-caryophyllene for test organisms: a – *Raphidocelis subcapitata* (microalgae, growth); b – *Lemna minor* (macrophyte, chlorophyll); c – *Lemna minor* (macrophyte, fresh weight); d – *Lemna minor* (macrophyte, number of fronds); e – *Daphnia* magna microcrustacean (immobility); f – *Panagrolaimus* sp (nematode, immobility); g – *Artemia salina* (microcrustacean, immobility). Red line: 50% confidence intervals (lower and upper); blue line: regression curve. HC5 = 0.026 mg/L.

## 4 Conclusion

The *in vitro* antibiosis test demonstrated the effectiveness of β-caryophyllene in inhibiting the growth of *Fusarium oxysporum* and inducing morphological changes. In contrast, graphene oxide did not show significant inhibitory effects on the fungus in the same assay. The combined application of β-caryophyllene and graphene oxide on cyclamen plants was more effective in both spraying and endotherapeutic applications, showing a synergistic effect. Graphene oxide may also act as an adsorbent for fusaric acid produced by Focy, contributing to enhanced antifungal activity. By improving plant quality and acting as a growth promoter, these agents have the potential to significantly increase the shelf life of cyclamen. Such applications can not only improve crop productivity but also help maintain quality over extended periods. Moreover, the determination of a risk parameter value (HC5) for the aquatic environment, using organisms from different trophic levels, supports the establishment of maximum permissible concentrations in water bodies associated with β-caryophyllene use. These findings highlight the potential of these compounds for plant protection and their contribution to sustainable agriculture.
